# Adolescent suicidal behaviours in 32 low- and middle-income countries

**DOI:** 10.2471/BLT.15.163295

**Published:** 2016-05-02

**Authors:** Britt McKinnon, Geneviève Gariépy, Mariane Sentenac, Frank J Elgar

**Affiliations:** a Institute for Health and Social Policy, McGill University, 1030 Pine Avenue West, Montreal, Quebec H3A 1A2, Canada.

## Abstract

**Objective:**

To estimate prevalence of suicidal ideation and suicidal ideation with a plan in each surveyed country and to examine cross-national differences in associated risk factors.

**Methods:**

We analysed data of students aged 13–17 years who participated in the 2003–2012 Global School-based Health Surveys in 32 countries, of which 29 are low- and middle-income. We used random effects meta-analysis to generate regional and overall pooled estimates. Multivariable logistic regression was used to estimate risk ratios for the associated risk factors. Population attributable fractions were estimated based on adjusted risk ratios and the prevalence of the determinants within each exposure level.

**Findings:**

Across all countries, the pooled 12-month prevalence of suicide ideation were 16.2% (95% confidence interval, CI: 15.6 to 16.7) among females and 12.2% (95% CI: 11.7 to 12.7) among males and ideation with a plan were 8.3% (95% CI: 7.9 to 8.7) among females and 5.8% (95% CI: 5.5 to 6.1) among males. Suicide ideation in the WHO Region of the Americas was higher in females than males, with an estimated prevalence ratio of 1.70 (95% CI: 1.60 to 1.81), while this ratio was 1.04 (95% CI: 0.98 to 1.10) in the WHO African Region. Factors associated with suicidal ideation in most countries included experiences of bullying and physical violence, loneliness, limited parental support and alcohol and tobacco use.

**Conclusion:**

The prevalence of adolescent suicidal behaviours varies across countries, yet a consistent set of risk factors of suicidal behaviours emerged across all regions and most countries.

## Résumé

**Objectif:**

Estimer la prévalence de l'idéation suicidaire et de l'idéation suicidaire avec un plan dans chacun des pays étudiés et examiner les différences entre les pays concernant les facteurs de risque associés.

**Méthodes:**

Nous avons analysé des données recueillies auprès d'élèves âgés de 13 à 17 ans ayant participé aux Enquêtes mondiales réalisées en milieu scolaire sur la santé des élèves entre 2003 et 2012 dans 32 pays, dont 29 à revenu faible et intermédiaire. Nous avons utilisé une méta-analyse à effets randomisés pour produire des estimations globales au niveau régional et général. Une régression logistique multivariée a été utilisée pour estimer les risques relatifs pour les facteurs de risque associés. Des fractions attribuables en population ont été estimées à partir des risques relatifs ajustés et de la prévalence des déterminants pour chaque niveau d'exposition.

**Résultats:**

Dans tous les pays, la prévalence globale de l'idéation suicidaire sur 12 mois était de 16,2% (intervalle de confiance, IC, à 95%: 15,6-16,7) pour les filles et de 12,2% (IC à 95%: 11,7-12,7) pour les garçons et la prévalence de l'idéation avec un plan était de 8,3% (IC à 95%: 7,9-8,7) pour les filles et de 5,8% (IC à 95%: 5,5-6,1) pour les garçons. Dans la Région OMS des Amériques, l'idéation suicidaire était plus importante chez les filles que chez les garçons, le taux de prévalence étant estimé à 1,70 (IC à 95%: 1,60-1,81) dans cette région contre 1,04 (IC à 95%: 0,98-1,10) dans la Région OMS de l’Afrique. Dans la plupart des pays, les facteurs associés à l'idéation suicidaire incluaient des cas de harcèlement et de violence physique, la solitude, un soutien parental limité et une consommation d'alcool et de tabac.

**Conclusion:**

Si la prévalence des comportements suicidaires des adolescents varie selon les pays, un ensemble cohérent de facteurs de risque associés aux comportements suicidaires est ressorti dans toutes les régions et dans la plupart des pays.

## Resumen

**Objetivo:**

Estimar la prevalencia de pensamientos suicidas y de pensamientos suicidas con un plan en los países encuestados y analizar las diferencias transversales de los factores de riesgo relacionados.

**Métodos:**

Se analizaron datos de estudiantes de edades comprendidas entre los 13 y 17 años que participaron en las Encuestas Mundiales de Salud a Escolares de 2003-2012 en 32 países, de los cuales 29 tienen ingresos bajos y medios. Se utilizó un metaanálisis de efectos aleatorios para generar estimaciones agrupadas generales y regionales. Se usó una regresión logística multivariable para calcular los coeficientes de riesgo relacionados con los factores de riesgo. Se calcularon fracciones atribuibles a la población según los coeficientes de riesgo ajustados y la prevalencia de los determinantes de cada nivel de exposición.

**Resultados:**

En todos los países, los resultados de la recopilación durante 12 meses de datos sobre la prevalencia de pensamientos suicidas fueron del 16,2% (intervalo de confianza, IC, del 95%: 15,6 a 16,7) entre las mujeres y del 12,2% (IC del 95%: 11,7 a 12,7) entre los hombres, y las cifras de pensamientos suicidas con un plan fueron del 8,3% (IC del 95%: 7,9 a 8,7) entre las mujeres y del 5,8% (IC del 95%: 5,5 a 6,1) entre los hombres. En la región de América de la OMS, las cifras de pensamientos suicidas eran mayores en mujeres que en hombres, con un coeficiente de prevalencia calculado del 1,70 (IC del 95%: 1,60 a 1,81), mientras que el mismo coeficiente fue del 1,04 (IC del 95%: 0,98 a 1,10) en la región africana de la OMS. En la mayoría de los países, los factores relacionados con los pensamientos suicidas incluían experiencias de acoso y violencia física, soledad, escaso apoyo de los padres y consumo de alcohol y tabaco.

**Conclusión:**

La prevalencia de las conductas suicidas de los adolescentes es diferente según el país, aunque ha surgido un conjunto de factores de riesgo de conducta suicida similares en todas las regiones y en la mayoría de los países.

## ملخص

**الغرض:**

تقدير مدى انتشار تكون الأفكار الانتحارية والأفكار الانتحارية التي تصاحبها خطة للتنفيذ في كل دولة خضعت لدراسة استقصائية بالإضافة إلى دراسة الفروق بين العديد من الدول فيما يتعلق بعوامل الخطورة ذات الصلة.

**الطريقة:**

أجرينا تحليلًا لبيانات الطلاب الذين تتراوح أعمارهم بين 13 و17 سنة ممن شاركوا في الدراسات الاستقصائية الصحية العالمية بالأوساط المدرسية في 32 دولة، حيث كانت 29 دولة من تلك الدول ضمن شريحة الدول منخفضة الدخل والدول متوسطة الدخل. استخدمنا نموذج الآثار العشوائية في التحليل التلوي لإيجاد تقديرات إقليمية وتقديرات عامة مجمعة. وتم استخدام التحوف اللوجيستي متعدد المتغيرات لتقدير نسب الخطورة المتعلقة بعوامل الخطورة ذات الصلة. وتم تقدير نسب الانخفاض المنسوبة إلى شريحة سكانية بناءً على نسب الخطورة المصححة ومدى انتشار المحددات في إطار كل مستوى للتعرض على حدة.

**النتائج:**

شهدت جميع البلدان انتشارًا لحالات تكون الأفكار الانتحارية على مدى 12 شهرًا وفقًا للتقديرات المجمعة بنسبة تبلغ 16.2% (بنسبة أرجحية مقدارها 95%: من 15.6 إلى 16.7) بين الإناث و12.2% (بنسبة أرجحية مقدارها 95%: من 11.7 إلى 12.7) بين الذكور، بينما بلغت نسبة تكون الأفكار الانتحارية التي تصاحبها خطة للتنفيذ 8.3% (بنسبة أرجحية مقدارها 95%: من 7.9 إلى 8.7) بين الإناث و5.8% (بنسبة أرجحية مقدارها 95%: من 5.5 إلى 6.1) بين الذكور. وزاد معدل تكون الأفكار الانتحارية في منطقة الأمريكتين (وفقًا لتقسيم منظمة الصحة العالمية) بين الإناث مقارنةً بالذكور، حيث بلغت نسبة انتشاره حسب التقديرات 1.70 (بنسبة أرجحية مقدارها 95%: من 1.60 إلى 1.81)، بينما بلغت تلك النسبة 1.04 (بنسبة أرجحية مقدارها 95%: من 0.98 إلى 1.10) في البلدان الأفريقية (وفقًا لتقسيم منظمة الصحة العالمية). وتضمنت العوامل المرتبطة بتكون الأفكار الانتحارية في معظم الدول التعرض لتجارب التسلط والعنف البدني، والشعور بالوحدة، وانخفاض مستوى الدعم الذي يمنحه الوالدان، وتعاطي الكحوليات والتبغ.

**الاستنتاج:**

يختلف مدى انتشار السلوك الانتحاري بين المراهقين فيما بين الدول، وبالرغم من ذلك ظهرت مجموعة من عوامل الخطورة المتماثلة في جميع المناطق ومعظم البلدان.

## 摘要

**目的:**

旨在评估各个受调查国家内的自杀意图发生率和有计划的自杀意图发生率，并且研究相关危险因素的跨国差异。

**方法:**

我们分析了参加 2003-2012 年在 32 个国家（其中 29 个为低收入和中等收入国家）开展的基于学校的全球性健康调查且年龄在 13-17 岁之间的学生数据。 我们采用了随机效应荟萃分析，以进行区域性和整体性综合评估。同时采用了多变量回归分析以评估相关风险因素的风险比率。 根据调整后的风险比率，评估了各个风险敞口等级内的群体可归因分率和决定因素发生率。

**结果:**

在所有国家，汇总 12 个月的女性自杀意图发生率为 16.2%（95% 置信区间，CI: 15.6 至 16.7），男性自杀意图发生率为 12.2%（95% CI: 11.7 至 12.7），女性间有计划的自杀意图发生率为 8.3%（95% CI: 7.9 至 8.7），男性间有计划的自杀意图发生率为 5.8%（95% CI: 5.5 至 6.1）。世界卫生组织美洲地区女性自杀意图发生率高于男性，估计发生率为 1.70（95% CI: 1.60 至 1.81），世界卫生组织非洲地区该比率为 1.04（95% CI: 0.98 至 1.10）。 在大多数国家中，与自杀意图有关的因素包括饱受欺凌与身体暴力、孤独、缺乏父母关爱以及饮酒吸烟的经历。

**结论:**

青少年自杀行为发生率因国家而异，然而在所有地区和大多数国家都存在一系列一致的自杀行为风险因素。

## Резюме

**Цель:**

Определить распространенность суицидальной направленности мышления и такой направленности с планированием суицида в каждой исследуемой стране и определить различия в связанных факторах риска между странами.

**Методы:**

Был проведен анализ данных об учащихся в возрасте от 13 до 17 лет, принявших участие в глобальных исследованиях состояния здоровья в школах 2003–2012 годов в 32 странах, 29 из которых относятся к странам с низким и средним уровнем доходов. С помощью метаанализа с использованием модели случайных эффектов были получены объединенные региональные и совокупные расчетные показатели. С помощью мультиномиальной логистической регрессии были подсчитаны отношения рисков для связанных факторов риска. Этиологические фракции были определены на основании скорректированных отношений рисков и распространенности определяющих факторов на каждом уровне воздействия.

**Результаты:**

Во всех вместе взятых странах объединенная годовая распространенность суицидальной направленности мышления составила 16,2% (95%-й доверительный интервал, ДИ: 15,6–16,7) среди женщин и 12,2% (95%-й ДИ: 11,7–12,7) среди мужчин, а распространенность такой направленности с планированием суицида составила 8,3% (95%-й ДИ: 7,9–8,7) среди женщин и 5,8% (95%-й ДИ: 5,5–6,1) среди мужчин. Суицидальная направленность мышления в странах Северной и Южной Америки, участвующих в деятельности ВОЗ, встречалась чаще среди женщин, чем среди мужчин; согласно подсчетам соотношение составило 1,70 (95%-й ДИ: 1,60–1,81), в то время как в странах Африки, участвующих в деятельности ВОЗ, этот же коэффициент составил 1,04 (95%-й ДИ: 0,98–1,10). В большинстве стран суицидальная направленность мышления была обусловлена такими факторами, как подверженность травле и физическому насилию, одиночество, недостаточная поддержка со стороны родителей, а также употребление алкоголя и табачных изделий.

**Вывод:**

Существуют различия в распространенности суицидального поведения подростков между странами, однако во всех регионах и в большинстве стран набор факторов риска суицидального поведения одинаков.

## Introduction

Worldwide, suicide accounts for an estimated 6% of all deaths among young people.^1^ As the second leading cause of mortality among females and the third leading cause among males aged 10–24 years, youth suicide is a major global public health concern.^1^ Low- and middle-income countries are home to more than 90% of the world’s children and youth and also account for over 75% of global suicide deaths.^2^ However, compared to high-income countries relatively little is known about the epidemiology of adolescent suicide and suicidal behaviours in low- and middle-income countries

Suicidal behaviours include ideation (thinking about killing oneself), planning suicide, attempting suicide and suicide itself.^2^ Suicidal ideation often emerges in adolescence and is prevalent among this age group, particularly among females.^3^ Across 17 European countries, the lifetime prevalence of ideation among students aged 15–16 years ranged from 15% (Armenia) to 31.5% (Hungary), while the lifetime prevalence of suicide attempts ranged from 4.1% to 23.5% in the same two countries, respectively.^4^ Across 49 low- and middle-income countries, 15.3% of adolescents aged 13–15 years had seriously considered suicide in the past year.^5^ Given that suicide ideation strongly and prospectively relates to suicide attempts and suicide,^6^^,^^7^ identifying potentially modifiable risk factors is essential for preventing these deaths.

Major risk factors for youth suicidal behaviours include being female, exposure to bullying and violence, alcohol and drug use, mental disorders and weak family and peer relationships.^4^^,^^8^^,^^9^ While much of this evidence comes from Europe and North America, recent research has expanded the knowledge of the determinants of youth suicidal behaviours in several low- and middle-income countries. Many factors associated with youth suicidal behaviours in low- and middle-income countries overlap with established risk factors from high-income countries, including bullying,^10^^,^^11^ physical and sexual abuse,^10^^,^^12^^,^^13^ mental disorders and depressive symptoms,^10^^,^^13^^–^^16^ substance use,^10^^,^^14^ and weak family and social relationships.^14^ However, research in some low- and middle-income countries suggests that gender and common mental health problems contribute less to suicidal behaviours.^14^^,^^17^^,^^18^ While studies from individual countries have provided insights about youth suicidal behaviours, differences in variable definitions and measures, study populations and analytical approaches make it difficult to compare the prevalence of and risk factors for youth suicidal behaviours across different settings.

The Global School-Based Health Survey (GSHS) has been conducted in over 80 countries worldwide and aims to provide comparable data on the health of adolescents aged 13–17 years^19^. GSHS data have been used to show cross-national variation in the prevalence of adolescent suicide ideation,^5^ examine suicide ideation in relation to psychosocial distress in seven African countries^20^ and study adolescent suicidal behaviours in individual low- and middle-income countries.^10^^,^^15^^,^^18^^,^^21^^,^^22^ In the present study, we estimate the cross-national prevalence of suicidal ideation and ideation with planning and examine correlates of these outcomes in a large and diverse sample of countries.

## Methods

### Data source

GSHS is a self-administered, school-based survey developed by the World Health Organization (WHO) and the United States Centers for Diseases Control and Prevention, in collaboration with the United Nations Children's Emergency Fund, the United Nations Educational, Scientific and Cultural Organization and the Joint United Nations Programme on HIV/AIDS.^19^^,^^23^ The survey uses standardized school-based sampling and a set of core questionnaire modules that address leading causes of morbidity and mortality worldwide, including alcohol and drug use, mental health, violence and unintentional injury and sexual behaviours. Some questions include country-specific examples, options or phrasing to facilitate adaptation of the surveys across diverse global populations. Many questions were adopted from the Youth Risk Behaviour Survey of American Adolescents, for which reliability has been formally evaluated.^24^ Reliability studies of the GSHS in low- and middle-income settings are limited, however one study among Fijian girls found high test–retest reliability of the two GSHS items on suicidal behaviours – suicidal ideation and ideation with a plan – with both showing agreement above 90% and kappa coefficients above 0.63.^25^

We included countries for which survey data on suicidal behaviours and potential risk factors were publicly available. Our sample included data from 38 surveys in 32 countries (Table 1). The survey dates ranged from 2003, the first year the survey was conducted, to 2012 the most recent year with publicly available data at the time of this analysis. The majority of countries conducted one survey over the time period. Where available, we pooled data from two surveys conducted in the same country. According to 2012 World Bank classification, 29 countries are considered low- and middle-income economies and three are high-income economies (Table 1).^26^ Although these three countries by definition are not low- and middle-income countries, we retained them for our analysis given the limited knowledge of adolescent suicidal behaviours in many high-income countries that are not part of the Organisation for Economic Co-operation and Development. Countries were grouped by WHO region. Because of the relatively small number of countries from the South-East Asia and Western Pacific Regions in our sample, data from these two regions were combined. Samples are nationally representative, except for the Bolivarian Republic of Venezuela, Chile, China, Ecuador, United Republic of Tanzania and Zimbabwe. For these countries, estimates are representative of selected cities or areas.

**Table 1:** Survey year(s) and sample size for countries that participated in the Global School-Based Health Survey,^19^ 2003–2012

Country by WHO region	Income classification^a^	Year of survey(s)	Sample size
**African**			
Benin	LIC	2009	2 659
Botswana	UMC	2005	2 114
Kenya	LIC	2003	3 317
Malawi	LIC	2009	2 213
Mauritania	LMC	2010	1 956
Uganda	LIC	2003	2 985
United Republic of Tanzania	LIC	2003	2 103
Zambia	LMC	2003	1 960
Zimbabwe	LIC	2003	5 482
**Americas**			
Argentina	UMC	2007	1 911
Chile	UMC	2004	8 028
Costa Rica	UMC	2009	2 626
Ecuador	UMC	2007	5 232
Guatemala	LMC	2009	5 370
Guyana	LMC	2004, 2010	3 471
Peru	UMC	2010	2 832
Trinidad and Tobago	HIC	2007, 2011	5 482
Venezuela (Bolivarian Republic of)	UMC	2003	4 252
**Eastern Mediterranean**			
Jordan	UMC	2004, 2007	4 359
Kuwait	HIC	2011	2 629
Lebanon	UMC	2007, 2011	7 245
Morocco	LMC	2006, 2010	5 275
Pakistan	LMC	2009	5 085
Tunisia	UMC	2008	2 759
United Arab Emirates	HIC	2005	15 077
**South-East Asia and Western Pacific**			
China	UMC	2004	8 753
Indonesia	LMC	2007	3 088
Malaysia	UMC	2012	20 849
Maldives	UMC	2009	2 919
Philippines	LMC	2007, 2011	17 497
Sri Lanka	LMC	2008	2 524
Thailand	UMC	2008	2 718

HIC: high-income countries; LIC: low-income; LMC: lower-middle income; UMC: Upper-middle income; WHO: World Health Organization.

^a^ Classification according to The World Bank.^26^

### Measures

The questionnaire contained two questions on suicidal ideation and planning where the response option was “yes” or “no” (Table 2). The questions were: “During the past 12 months, did you ever seriously consider attempting suicide?” and: “During the past 12 months, did you ever make a plan about how you would attempt suicide?” Consistent with a recent study using the 2009 Benin GSHS, we defined variables for suicidal ideation as responding “yes” to the first question and variables for suicidal ideation with planning as responding “yes” to both questions.^14^

**Table 2:** Global School-Based Health Survey^19^ questions used in the analysis of adolescent suicidal behaviours in low- and middle-income countries

Variable	Question	Values
Suicidal ideation	During the past 12 months, did you ever seriously consider attempting suicide?	0 = no 1 = yes
Suicidal planning	During the past 12 months, did you ever make a plan about how you would attempt suicide?	0 = no 1 = yes
Physical attack^a^	During the past 12 months, how many times were you physically attacked?	1 = 0 times 2 = 1 time 3 = 2 or more times
Bullying^b^	During the past 30 days, on how many days were you bullied?	1 = 0 days 2 = 1 or 2 days 3 = 3 or more days
Food insecurity	During the past 30 days, how often did you go hungry because there was not enough food in your home?	1 = never 2 = sometimes/rarely 3 = most of the time/always
Loneliness	During the past 12 months, how often have you felt lonely?	1 = never/rarely 2 = sometimes 3 = most of the time/always
Lack of parental support	During the past 30 days, how often did your parents or guardians understand your problems and worries?	1 = most of the time/always 2 = sometimes 3 = never/rarely
Few close friends	How many close friends do you have?	1 = 3 or more 2 = 1 or 2 3 = none
Alcohol use^c^	During the past 30 days, on how many days did you have at least one drink containing alcohol?	1 = 0 days 2 = 1–2 days 3 = 3 or more days
Cigarette smoking	During the past 30 days, on how many days did you smoke cigarettes?	1 = 0 days 2 = 1–5 days 3 = 6 or more days

^a^ In the survey questionnaire physical attack is defined as “when one or more people hit or strike someone, or when one or more people hurt another person with a weapon (such as a stick, knife, or gun)”. The survey questionnaire specifies that “it is not a physical attack when two students of about the same strength or power choose to fight each other”.

^b^ Bullying is defined as “when a student or group of students say or do bad and unpleasant things to another student” or “when a student is teased a lot in an unpleasant way” or “when a student is left out of things on purpose”. The survey questionnaire specifies that “it is not bullying when two students of about the same strength or power argue or fight or when teasing is done in a friendly and fun way”.

^c^ Drinking alcohol also includes consuming locally produced alcoholic drinks. The survey questionnaire specifies that “drinking alcohol does not include drinking a few sips of wine for religious purposes. A drink is defined as a glass of wine, a bottle of beer, a small glass of liquor, or a mixed drink”.

We selected a priori potential risk factors for suicidal ideation based on previous research on adolescents in low- , middle- and high-income countries and their inclusion in the survey.^27^^–^^30^ The determinants included were: psychosocial symptoms (loneliness, having close friends and parental support); substance use (alcohol use and cigarette smoking); being physically attacked; and bullying victimization. Given the survey does not include questions on family socioeconomic conditions, we included information on the frequency of going to bed hungry to capture some socioeconomic-related variation. The survey in some countries asked about other potentially important determinants (e.g. drug use, sexual assault); these were excluded because they were missing > 50% of the responses across the surveys.

Our analysis included an initial sample size of 164 770 across the 32 countries. The percentages of missing data were 1.8% (2966) for suicide ideation and 2.7% (4449) for planning suicide. Other variables were missing less than 5%, except for bullying (12.0%, 19 772), smoking (15.1%, 24 880), physical attacks (26.8%, 44 158) and alcohol use (28.4%, 46 795). To account for missing data, we performed multiple imputation using the mi impute chained procedure in Stata version 12 (StataCorp. LP, College Station, United States of America), which uses an iterative multivariable regression procedure to generate distributions for each variable with missing data that are conditional on all other variables in the imputation models.^31^ All variables with missing data were imputed using appropriate distributions to model each variable, for example logistic, ordered logistic regressions. A total of 10 imputed data sets were generated. Results were pooled across imputed data sets using Stata’s mi estimate procedures.

### Statistical analysis

We estimated the prevalence of suicidal ideation and ideation with a plan among males and females in each country using age-adjusted logistic regression to facilitate comparability of estimates across countries.^32^ Ratios and differences comparing the prevalence of suicidal behaviours for females compared to males were estimated for each country. Random effects meta-analysis was used to generate regional and overall pooled estimates, using the DerSimonian and Laird inverse-variance method.^33^ We used meta-regression to correlate country-level estimates of adolescent suicidal ideation with estimated national mortality rates from self-harm among young people aged 15–29 years.^2^^,^^34^

Multivariable logistic regression was used to estimate risk ratios (RR) and 95% confidence intervals (CI) measuring associations between the determinants and suicidal behaviours. RRs were calculated from average marginal probabilities estimated from the logistic coefficients.^32^ Multivariable models were estimated separately by WHO region and included fixed effects for country and survey year. Population attributable fractions (PAF) were estimated for the risk factors, based on adjusted risk ratios and the prevalence of the determinants within each exposure level.^35^^,^^36^ All analyses incorporated sampling weights where available and accounted for clustering at the school level.

## Results

The pooled 12-month prevalence of suicide ideation for females was 16.2% (95% CI: 15.6 to 16.7) and for males 12.2% (95% CI: 11.7 to 12.7). For suicide ideation with a plan the pooled 12-month prevalence for females was 8.3% (95% CI: 7.9 to 8.7) and for males 5.8% (95% CI: 5.5 to 6.1). There was considerable heterogeneity between countries in the prevalence of suicide ideation, ranging from 5.1% (95% CI: 2.1 to 8.1) in Indonesia to 28.1% (95% CI: 22.5 to 33.7) in Zambia. For ideation with a plan for both sexes the prevalence ranged from 1.7% (95% CI: −0.1 to 3.5) in the United Republic of Tanzania to 15.3% (95% CI: 11.6 to 19.0) in Benin and Kenya 15.3% (95% CI: 12.6 to 18.1). The African Region showed the highest overall pooled prevalence of suicide ideation (21.6%; 95% CI: 20.4 to 22.9) and no evidence of gender differences. By contrast, suicide ideation in the Region of the Americas was markedly higher in females than males, with an estimated prevalence ratio of 1.7 (95% CI: 1.6 to 1.8). The South-East Asia Region and Western Pacific Region had a relatively low prevalence of suicidal behaviours for both sexes, 10.7% (95% CI: 9.9 to 11.5) for ideation and 5.0% (95% CI: 4.5 to 5.4) for ideation with a plan (Table 3; available at: http://www.who.int/bulletin/volumes/94/5/15-163295).

**Table 3:** Prevalence of suicide ideation and suicide ideation with a plan by country, 2003–2012

Country by WHO region	Suicide ideation				Suicide ideation with a plan			
	Prevalence, % (95% CI)		Female/male ratio (95% CI)	Female/Male Difference (95% CI)	Prevalence, % (95% CI)		Female/Male Ratio (95% CI)	Female/Male Difference (95% CI)
	Females	Males			Females	Males		
**African**								
Benin	21.4 (17.3 to 25.5)	21.7 (17.5 to 25.9)	0.99 (0.86 to 1.12)	−0.3 (−3.2 to 2.5)	14.4 (11.1 to 17.8)	15.7(11.7 to 19.7)	0.92 (0.79 to 1.05)	−1.3 (−3.5 to 0.9)
Botswana	21.9 (17.9 to 26.0)	19.7 (15.6 to 23.8)	1.11 (0.94 to 1.29)	2.2 (−1.0 to 5.5)	11.4 (8.4 to 17.8)	9.5 (6.4 to 12.7)	1.20 (0.92 to 1.48)	1.9 (−0.4 to 4.3)
Kenya	27.8 (24.4 to 31.2)	27.2 (21.5 to 32.8)	1.02 (0.83 to 1.22)	0.6 (−4.5 to 5.8)	16.0 (12.7 to 19.3)	14.6 (11.8 to 17.5)	1.09 (0.88 to 1.31)	1.3 (−1.7 to 4.4)
Malawi	11.5 (6.8 to 16.3)	9.4 (4.2 to 14.5)	1.23 (0.68 to 1.79)	2.2 (−2.3 to 6.6)	5.9 (2.4 to 9.3)	4.8 (2.5 to 7.2)	1.22 (0.45 to 1.98)	1.0 (−2.4 to 4.5)
Mauritania	19.3 (13.6 to 25.0)	20.1 (13.4 to 26.8)	0.96 (0.72 to 1.20)	−0.8 (−5.8 to 4.1)	9.5 (5.4 to 13.5)	11.1 (7.7 to 14.6)	0.85 (0.54 to 1.17)	−1.6 (−5.3 to 2.0)
Uganda	23.0 (19.3 to 26.7)	17.7 (13.9 to 21.5)	1.30 (1.06 to 1.54)	5.3 (1.7 to 8.9)	13.6 (10.3 to 17.0)	10.7 (7.3 to 14.0)	1.28 (0.93 to 1.62)	2.9 (−0.2 to 6.1)
United Republic of Tanzania	7.6 (3.1 to 12.1)	8.4 (3.5 to 13.4)	0.90 (0.69 to 1.11)	−0.8 (−2.7 to 1.0)	1.7 (−0.0 to 3.5)	1.6 (−0.2 to 3.5)	1.06 (0.70 to 1.41)	0.1 (−0.4 to 0.6)
Zambia	28.2 (22.7 to 33.7)	28 (21.5 to 34.6)	1.01 (0.84 to 1.17)	0.2 (−4.6 to 4.9)	12.6 (8.9 to 16.4)	11.5 (7.5 to 15.5)	1.10 (0.83 to 1.36)	1.1 (−1.7 to 3.9)
Zimbabwe	27.9 (25.1 to 30.6)	25.5 (22.4 to 28.6)	1.09 (0.96 to 1.23)	2.4 (−0.9 to 5.6)	14.4 (12.3 to 16.5)	11.6 (9.3 to 13.9)	1.25 (1.01 to 1.48)	2.8 (0.5 to 5.2)
Region Pooled	22.5 (21.2 to 23.8)	20.1 (18.6 to 21.6)	1.04 (0.98 to 1.10)	0.8 (−0.3 to 1.9)	9.6 (8.7 to 10.5)	8.4 (7.4 to 9.3)	1.05 (0.97 to 1.13)	0.3 (−0.1 to 0.8)
**Americas**								
Argentina	19.7 (14.5 to 24.9)	13.7 (9.4 to 18.0)	1.44 (1.04 to 1.84)	6.0 (1.5 to 10.6)	10.7 (7.1 to 14.4)	7.4 (3.8 to 10.9)	1.45 (0.95 to 1.95)	3.3 (0.5 to 6.2)
Chile	32.1 (26.4 to 37.8)	13.7 (10.4 to 17.0)	2.35 (1.98 to 2.72)	18.4 (14.5 to 22.3)	20.9 (16.5 to 25.3)	7.5 (5.5 to 9.5)	2.79 (2.28 to 3.30)	13.4 (10.2 to 16.6)
Costa Rica	12.6 (9.8 to 15.3)	6.7 (4.5 to 8.9)	1.87 (1.41 to 2.33)	5.8 (3.7 to 8.0)	5.3 (3.2 to 7.4)	3.2 (1.7 to 4.6)	1.68 (1.15 to 2.21)	2.1 (0.7 to 3.6)
Ecuador	23.2 (17.7 to 28.8)	14.4 (11.1 to 17.7)	1.61 (1.33 to 1.89)	8.8 (5.0 to 12.7)	17.1 (12.4 to 21.8)	10.5 (7.8 to 13.2)	1.63 (1.33 to 1.93)	6.6 (3.4 to 9.8)
Guatemala	17.7 (15.2 to 20.1)	10.1 (8.4 to 11.7)	1.75 (1.42 to 2.09)	7.6 (4.9 to 10.3)	12.7 (10.3 to 15.0)	5.9 (4.5 to 7.3)	2.14 (1.63 to 2.66)	6.8 (4.5 to 9.0)
Guyana	24.9 (21.7 to 28.1)	15.0 (12.1 to 18.0)	1.66 (1.35 to 1.96)	9.9 (6.6 to 13.1)	15.9 (13.4 to 18.3)	8.5 (6.3 to 10.7)	1.87 (1.43 to 2.31)	7.4 (4.9 to 9.8)
Peru	27.3 (23.1 to 31.5)	11.9 (9.6 to 14.3)	2.29 (1.92 to 2.66)	15.4 (12.0 to 18.7)	17.6 (14.6 to 20.6)	6.2 (4.8 to 7.7)	2.82 (2.24 to 3.40)	11.3 (8.8 to 13.9)
Trinidad and Tobago	22.8 (18.7 to 26.9)	15.3 (11.5 to 19.1)	1.49 (1.20 to 1.78)	7.5 (4.1 to 10.9)	14.8 (11.6 to 18.0)	9.8 (6.6 to 13.0)	1.50 (1.12 to 1.89)	4.9 (2.2 to 7.7)
Venezuela (Bolivarian Republic of)	17.3 (12.0 to 22.5)	12.3 (9.4 to 15.3)	1.40 (1.13 to 1.67)	4.9 (1.4 to 8.4)	12.1 (8.1 to 16.1)	7.4 (4.8 to 10.1)	1.63 (1.17 to 2.08)	4.7 (1.7 to 7.6)
Region Pooled	20.1 (18.9 to 21.3)	11.4 (10.5 to 12.3)	1.70 (1.60 to 1.81)	8.7 (7.7 to 9.8)	12.8 (11.8 to 13.8)	6.4 (5.8 to 7.1)	1.84 (1.69 to 2.00)	5.6 (4.8 to 6.4)
**Eastern Mediterranean**								
Jordan	16.6 (13.8 to 19.4)	13.7 (11.4 to 16.0)	1.21 (0.99 to 1.44)	2.9 (0.1 to 5.7)	10.0 (8.1 to 12.0)	7.2 (5.5 to 8.9)	1.40 (1.06 to 1.74)	2.9 (0.8 to 4.9)
Kuwait	19.0 (16.4 to 21.6)	16.3 (11.7 to 20.9)	1.17 (0.83 to 1.50)	2.7 (−2.1 to 7.6)	10.8 (9.2 to 12.3)	10.0 (6.7 to 13.4)	1.07 (0.72 to 1.43)	0.7 (−2.6 to 4.1)
Lebanon	20.5 (17.4 to 23.5)	15.3 (12.6 to 18.0)	1.34 (1.13 to 1.54)	5.1 (2.5 to 7.8)	12.0 (8.9 to 15.1)	8.7 (6.6 to 13.4)	1.37 (1.04 to 1.70)	3.3 (0.6 to 5.9)
Morocco	20.4 (16.7 to 24.1)	14 (11.5 to 16.6)	1.45 (1.24 to 1.66)	6.3 (3.6 to 9.1)	11.4 (8.9 to 15.1)	7.5 (5.6 to 9.4)	1.53 (1.26 to 1.81)	4.0 (2.2 to 5.7)
Pakistan	7.5 (5.1 to 9.9)	8.3 (6.5 to 10.1)	0.90 (0.61 to 1.19)	−0.8 (−3.3 to 1.7)	4.6 (2.1 to 8.1)	4.6 (3.4 to 5.9)	0.99 (0.50 to 1.49)	0.0 (−2.3 to 2.2)
Tunisia	25.8 (21.1 to 30.6)	18.9 (15.2 to 22.6)	1.37 (1.11 to 1.63)	7.0 (2.7 to 11.2)	13.7 (9.7 to 17.6)	10.4 (7.3 to 13.6)	1.31 (0.92 to 1.70)	3.2 (−0.4 to 6.9)
United Arab Emirates	14.0 (11.3 to 16.7)	14.4 (11.5 to 16.6)	0.97 (0.83 to 1.11)	−0.4 (−2.4 to 1.6)	7.6 (5.9 to 9.3)	7.5 (6.0 to 9.0)	1.02 (0.83 to 1.21)	0.1 (−1.3 to 1.5)
Region Pooled	16.0 (14.9 to 17.1)	12.9 (12.0 to 13.9)	1.18 (1.10 to 1.26)	2.5 (1.4 to 3.5)	9.5 (8.7 to 10.3)	7.0 (6.3 to 7.6)	1.22 (1.11 to 1.33)	1.8 (1.0 to 2.6)
**South-East Asia and Western Pacific**								
								
China	17.6 (14.9 to 20.3)	14.0 (11.4 to 16.5)	1.26 (1.11 to 1.41)	3.7 (1.9 to 5.4)	7.1 (5.9 to 8.3)	5.1 (3.9 to 6.2)	1.40 (1.08 to 1.73)	2.0 (0.7 to 3.4)
Indonesia	6.1 (2.4 to 9.8)	4.1 (1.5 to 6.7)	1.50 (0.92 to 2.08)	2.0 (−0.2 to 4.3)	3.5 (1.4 to 5.6)	2.1 (0.7 to 3.5)	1.64 (1.03 to 2.25)	1.4 (0.1 to 2.6)
Malaysia	9.6 (8.4 to 10.7)	6.7 (5.2 to 8.2)	1.43 (1.14 to 1.72)	2.9 (1.4 to 4.3)	4.7 (3.9 to 5.5)	3.3 (2.5 to 4.1)	1.43 (1.06 to 1.79)	1.4 (0.4 to 2.4)
Maldives	17.5 (13.8 to 21.2)	17.2 (13.1 to 21.4)	1.01 (0.83 to 1.20)	0.2 (−2.9 to 3.4)	10.4 (7.3 to 13.5)	9.3 (6.4 to 12.2)	1.12 (0.87 to 1.37)	1.1 (−1.1 to 3.3)
Philippines	23.2 (20.4 to 25.9)	13.9 (11.9 to 16.0)	1.66 (1.45 to 1.87)	9.2 (6.9 to 11.5)	9.4 (7.8 to 11.1)	5.0 (4.1 to 6.0)	1.88 (1.57 to 2.19)	4.4 (3.1 to 5.8)
Sri Lanka	9.9 (6.9 to 12.8)	12 (8.2 to 15.8)	0.82 (0.56 to 1.08)	−2.2 (−5.7 to 1.4)	3.9 (1.4 to 6.5)	4.3 (2.0 to 6.7)	0.91 (0.39 to 1.43)	−0.4 (−2.7 to 2.0)
Thailand	7.0 (4.1 to 9.9)	8.9 (6.3 to 11.4)	0.78 (0.52 to 1.04)	−1.9 (−4.4 to 0.5)	4.2 (1.9 to 6.5)	4.7 (2.5 to 6.8)	0.90 (0.55 to 1.26)	−0.4 (−2.1 to 1.2)
Region Pooled	11.7 (10.9 to 12.6)	9.6 (8.7 to 10.5)	1.20 (1.11 to 1.28)	2.7 (1.9 to 5.4)	5.8 (5.3 to 6.4)	4.1 (3.7 to 4.6)	1.32 (1.19 to 1.46)	1.7 (1.2 to 2.2)
**All regions**								
Overall Pooled	16.2 (15.6 to 16.7)	12.2 (11.7 to 12.7)	1.19 (1.16 to 1.23)	3.6 (3.1 to 4.1)	8.3 (7.9 to 8.7)	5.8 (5.5 to 6.1)	1.24 (1.19 to 1.30)	1.7 (1.4 to 2.0)

CI: confidence interval; WHO: World Health Organization.

Notes: Pooled estimates are from random effects meta-analysis, for which the inverse-variance DerSimonian and Laird method was used. Inconsistencies arise in some values due to rounding.

Data source: Global School-Based Health Survey.^19^

**Fig. 1:**
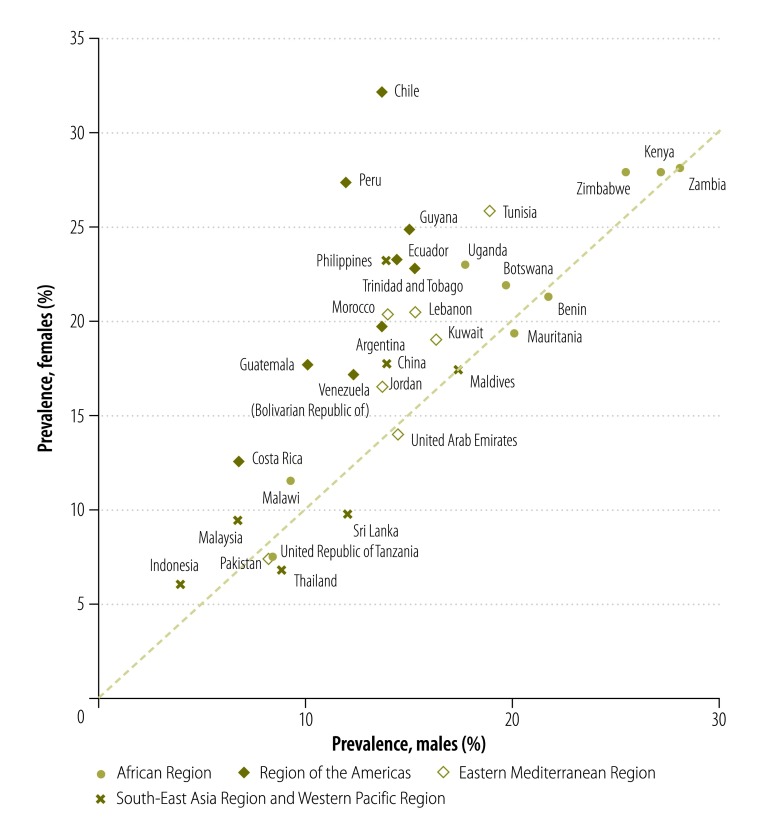
Prevalence of suicide ideation in the past 12 months among male and female students in WHO regions, 2003–2012

**Fig. 2:**
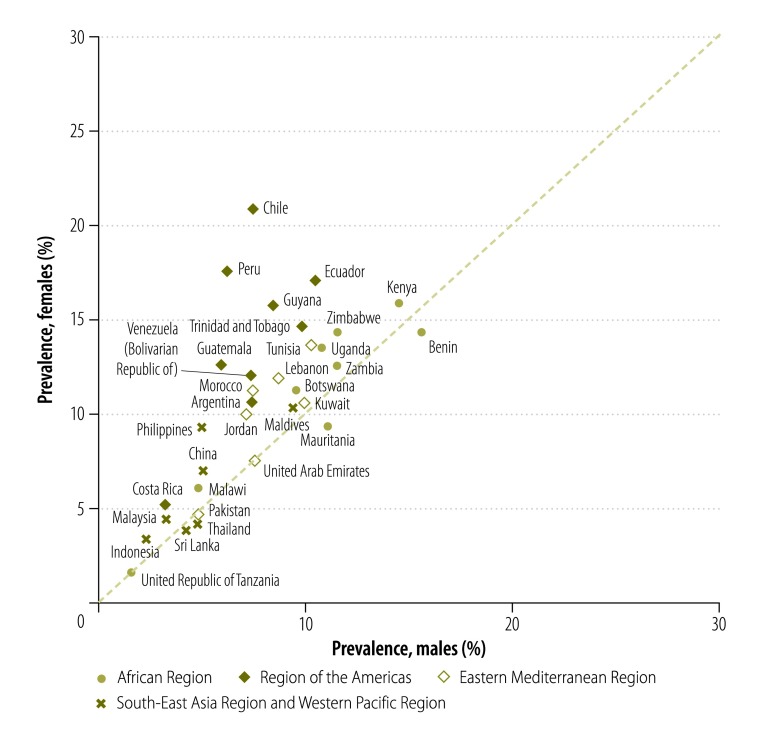
Prevalence of suicide ideation with a plan in the past 12 months among male and female students in WHO regions, 2003–2012

**Fig. 3:**
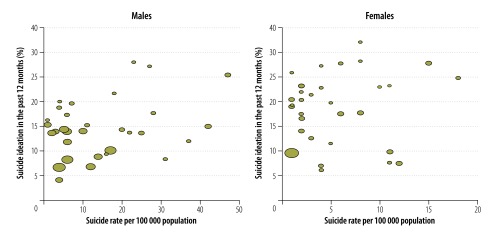
Meta-regression plots of the association between suicide ideation and national suicide rates, 2012

Fig. 1 and Fig. 2 summarize the country-specific predicted prevalence estimates of suicide ideation and ideation with a plan for males and females. Approximately half the countries have statistically significant gender inequality. In all countries with gender differences, suicidal behaviours were more common in females than in males. Correlations between youth suicidal ideation and national mortality rates from self-harm were weak to moderate and stronger among males (*r* = 0.29) than among females (*r* = 0.11; Fig. 3). Among the eight explored determinants, the highest correlations were between smoking and drinking (*r* = 0.31) and having been physically attacked and bullied (*r* = 0.27) and suicide ideation.

WHO: World Health Organization.

Notes: The dashed diagonal line indicates gender equality in the prevalence of suicidal behaviours. Pearson’s correlation coefficients for the association between male and female prevalence of suicide ideation and ideation with a plan are 0.74 and 0.70, respectively.

WHO: World Health Organization.

Notes: The dashed diagonal line indicates gender equality in the prevalence of suicidal behaviours. Pearson’s correlation coefficients for the association between male and female prevalence of suicide ideation and ideation with a plan are 0.74 and 0.70, respectively.

CI: confidence interval.

Notes: Mortality rates from self-harm for persons aged 15–29 years for the year 2012 obtained from the World Health Organization Department of Health Statistics and Information Systems. Meta-regression coefficients for males: β = 0.13 (95% CI: −0.03 to 0.30); and females: β = 0.17 (95% CI: −0.42 to 0.76). The size of the bubble represents the precision of the study for each country.

Data source: World Health Organization.^34^

Table 4 (available at: http://www.who.int/bulletin/volumes/94/5/15-163295) presents adjusted risks and risk ratios for suicide ideation during the past 12 months, stratified by WHO region. With the exception of age, all determinants correlated independently to suicidal ideation in all regions. Moreover, higher RRs were almost always observed for the more extreme categories of the determinants. Determinants of suicide ideation appeared generally consistent across regions, with loneliness, alcohol use and bullying showing stronger associations. Across all regions, the adjusted risk of suicidal ideation among students who answered “mostly/always feeling lonely” was greater than 10 percentage points above those answering “never/rarely feeling lonely,” for example 30.7% versus 20.3% for the African Region and 31.9% versus 12.3% for the Region of the Americas. Analysis for the outcome of suicide ideation with a plan obtained similar results (Table 5; available at: http://www.who.int/bulletin/volumes/94/5/15-163295). 

**Table 4:** Risk factors associated with suicide ideation during the past 12 months by WHO region, 2003–2012

Characteristics	African Region			Region of the Americas			Eastern Mediterranean Region			South-East Asia and Western Pacific Regions			
	No. (% in sample) *n* = 24 789	Risk^a^	RR (95% CI)^a^	No. (% in sample) *n* = 39 204	Risk^a^	RR (95% CI)^a^	No. (% in sample) *n* = 58 348	Risk^a^	RR (95% CI)^a^	No. (% in sample) *n* = 42 429	Risk^a^	RR (95% CI)^a^	
**Sex**												
Male	12 543 (50.6)	21.0	Ref	19 563 (49.9)	12.5	Ref	30 341 (52.0)	12.5	Ref	21 172 (49.9)	9.9	Ref	
Female	12 246 (49.4)	25.0	1.19 (1.12 to 1.26)	19 641 (50.1)	21.3	1.70 (1.59 to 1.82)	28 007 (48.0)	16.7	1.35 (1.27 to 1.44)	21 257 (50.1)	14.2	1.44 (1.34 to 1.55)		
**Age**															
< 12 years	1 859 (7.5)	23.0	1.01 (0.86 to 1.15)	5 489 (14.0)	17.2	1.00 (0.91 to 1.10)	5 601 (9.6)	12.5	0.88 (0.79 to 0.97)	3 225 (7.6)	12.5	1.04 (0.90 to 1.18)		
13 years	3 644 (14.7)	21.8	0.95 (0.86 to 1.04)	9 644 (24.6)	16.9	0.99 (0.93 to 1.05)	12 253 (21.0)	13.3	0.90 (0.83 to 0.97)	10 310 (24.3)	11.0	0.91 (0.83 to 0.99)		
14 years	5 949 (24.0)	22.9	Ref	10 663 (27.2)	17.2	Ref	16 863 (28.9)	14.4	Ref	12 050 (28.4)	12.0	Ref		
15 years	6 544 (26.4)	22.7	1.00 (0.93 to 1.07)	8 703 (22.2)	16.5	0.96 (0.89 to 1.03)	15 404 (26.4)	15.0	1.01 (0.93 to 1.09)	9 971 (23.5)	12.0	1.00 (0.92 to 1.08)		
16+ years	6 817 (27.5)	23.7	1.04 (0.95 to 1.12)	4 704 (12.0)	16.7	0.97 (0.89 to 1.06)	8 169 (14.0)	16.2	1.11 (1.01 to 1.21)	6 873 (16.2)	12.7	1.06 (0.96 to 1.16)		
**Attacked in past 12 months**														
Never	12 543 (50.6)	20.1	Ref	25 953 (66.2)	14.8	Ref	37 051(63.5)	12.9	Ref	28 258 (66.6)	10.6	Ref		
Once	4 239 (17.1)	22.9	1.14 (1.03 to 1.25)	5 489 (14.0)	18.8	1.27 (1.16 to 1.38)	8 927 (15.3)	15.3	1.13 (1.03 to 1.22)	5 558 (13.1)	13.0	1.24 (1.11 to 1.36)		
≥ 2 times	8 007 (32.3)	26.7	1.33 (1.22 to 1.45)	7 762 (19.8)	21.2	1.43 (1.33 to 1.54)	12 370 (21.2)	17.6	1.35 (1.26 to 1.44)	8 613 (20.3)	14.8	1.41 (1.30 to 1.52)		
**Bullied in past 30 days**														
Never	12 419 (50.1)	19.2	Ref	26 384 (67.3)	14.7	Ref	40 318 (69.1)	12.3	Ref	29 191 (68.8)	9.9	Ref		
1–2 days	6 346 (25.6)	24.3	1.27 (1.18 to 1.36)	7 214 (18.4)	19.0	1.29 (1.21 to 1.38)	11 028 (18.9)	16.8	1.36 (1.25 to 1.47)	7 934 (18.7)	14.5	1.46 (1.35 to 1.57)		
≥ 3 days	6 024 (24.3)	27.8	1.45 (1.35 to 1.54)	5 606 (14.3)	22.0	1.50 (1.39 to 1.61)	7 002 (12.0)	21.3	1.71 (1.56 to 1.86)	5 304 (12.5)	16.4	1.65 (1.50 to 1.80)		
**Went hungry in past 30 days**														
Never	9 445 (38.1)	21.4	Ref	25 875 (66.0)	16.6	Ref	35 651 (61.1)	13.8	Ref	20 154 (47.5)	11.2	Ref		
Sometimes/rarely	11 973 (48.3)	23.5	1.10 (1.03 to 1.16)	11 879 (30.3)	17.0	1.02 (0.97 to 1.08)	19 547 (33.5)	14.5	1.04 (0.98 to 1.11)	20 239 (47.7)	12.2	1.09 (1.03 to 1.16)		
Mostly/always	3 371 (13.6)	24.6	1.15 (1.05 to 1.26)	1 451 (3.7)	19.3	1.16 (1.02 to 1.30)	3 676 (6.3)	17.5	1.23 (1.12 to 1.34)	2 037 (4.8)	15.1	1.35 (1.21 to 1.49)		
**Number of close friends**														
Three or more	9 222 (37.2)	21.1	Ref	25 639 (65.4)	15.6	Ref	35 125 (60.2)	12.9	Ref	31 016 (73.1)	11.2	Ref		
One or two	12 568 (50.7)	22.9	1.09 (1.02 to 1.15)	10 781 (27.5)	18.0	1.16 (1.09 to 1.22)	19 547 (33.5)	15.5	1.20 (1.12 to 1.28)	9 292 (21.9)	13.0	1.17 (1.09 to 1.25)		
None	2 975 (12.09)	28.6	1.36 (1.25 to 1.47)	2 783 (7.1)	22.9	1.47 (1.34 to 1.59)	3 676 (6.3)	22.5	1.73 (1.57 to 1.89)	2 121 (5.0)	15.9	1.42 (1.27 to 1.58)		
**Lonely in past 12 months**														
Never/rarely	12 370 (49.9)	20.3	Ref	23 914 (61.0)	12.3	Ref	37 051 (63.5)	11.5	Ref	24 948 (58.8)	9.4	Ref		
Sometimes	8 577 (34.6)	22.7	1.12 (1.04 to 1.20)	10 781 (27.5)	17.8	1.45 (1.35 to 1.54)	12 428 (21.3)	14.7	1.30 (1.20 to 1.40)	13 365 (31.5)	12.2	1.30 (1.22 to 1.39)		
Mostly/always	4 115 (16.6)	30.7	1.51 (1.39 to 1.63)	4 508 (11.5)	31.9	2.58 (2.40 to 2.77)	8 811 (15.1)	23.2	2.04 (1.90 to 2.18)	4 073 (9.6)	21.9	2.34 (2.12 to 2.55)		
**Parental understanding**														
Mostly/always	9 692 (39.1)	20.3	Ref	17 171 (43.8)	13.3	Ref	25 615 (43.9)	11.3	Ref	15 274 (36.0)	9.2	Ref		
Sometimes	5 999 (24.2)	22.5	1.11 (1.03 to 1.18)	7 449 (19.0)	15.7	1.18 (1.08 to 1.28)	10 269 (17.6)	13.7	1.22 (1.12 to 1.33)	10 565 (24.9)	10.6	1.16 (1.05 to 1.26)		
Never/rarely	9 098 (36.7)	26.0	1.28 (1.19 to 1.37)	14 584 (37.2)	20.8	1.57 (1.47 to 1.67)	22 464 (38.5)	17.8	1.54 (1.44 to 1.64)	16 590 (39.1)	14.7	1.60 (1.48 to 1.72)		
**Smoking in past 30 days**														
0 days	22 211 (89.6)	22.0	Ref	32 892 (83.9)	15.6	Ref	52 513 (90.0)	13.4	Ref	38 101 (89.8)	11.4	Ref		
1–5 days	1 487 (6.0)	23.5	1.31 (1.15 to 1.48)	3 803 (9.7)	20.4	1.30 (1.17 to 1.44)	3 209 (5.5)	20.6	1.50 (1.34 to 1.66)	2 461 (5.8)	15.8	1.38 (1.24 to 1.52)		
≥ 6 days	1 091 (4.4)	24.6	1.39 (1.22 to 1.56)	2 548 (6.5)	24.3	1.56 (1.40 to 1.71)	2 626 (4.5)	22.0	1.61 (1.39 to 1.83)	1 824 (4.3)	15.1	1.32 (1.14 to 1.50)		
**Drink alcohol in past 30 days**														
0 days	19 509 (78.7)	21.0	Ref	26 855 (68.5)	14.2	Ref	47 554 (81.5)	13.0	Ref	37 380 (88.1)	10.9	Ref		
1–2 days	2 876 (11.6)	27.4	1.31 (1.18 to 1.44)	7 606 (19.4)	20.4	1.44 (1.34 to 1.54)	6 535 (11.2)	18.7	1.39 (1.27 to 1.51)	3 352 (7.9)	16.8	1.54 (1.41 to 1.67)		
≥ 3 days	2 380 (9.6)	30.9	1.47 (1.35 to 1.60)	4 744 (12.1)	23.7	1.67 (1.53 to 1.81)	4 259 (7.3)	20.9	1.68 (1.50 to 1.86)	1 697 (4.0)	18.5	1.69 (1.50 to 1.88)		

CI: confidence interval; Ref: Reference group; RR: Relative risk; WHO: World Health Organization.

^a^ Predicted risks of suicide ideation and RRs were estimated from multivariable logistic regression models that additionally include fixed effects for country and survey year.

Note: Inconsistencies arise in some values due to rounding.

Data source: Global School-Based Health Survey.^19^

**Table 5:** Risk factors associated with suicide ideation with a plan during the past 12 months by WHO region, 2003–2012

Characteristics	African Region		Region of the Americas		Eastern Mediterranean Region			South-East Asia and Western Pacific Regions	
	Predicted risk^a^	RR (95% CI)^a^	Predicted risk^a^	RR (95% CI)^a^	Predicted risk^a^	RR (95% CI)^a^	Predicted risk^a^		RR (95% CI)^a^
**Sex**									
Male	9.0	Ref	7.1	Ref	6.9	Ref		3.9	Ref
Female	10.8	1.19 (1.08 to 1.30)	13.3	1.88 (1.72 to 2.04)	9.8	1.42 (1.31 to 1.54)		6.3	1.62 (1.44 to 1.80)
**Age, years**									
< 12 years	9.9	1.03 (0.77 to 1.28)	9.4	0.89 (0.76 to 1.01)	6.8	0.82 (0.70 to 0.93)		5.1	1.01 (0.82 to 1.20)
13 years	9.0	0.93 (0.79 to 1.07)	10.2	0.96 (0.88 to 1.03)	6.9	0.82 (0.73 to 0.91)		4.4	0.89 (0.78 to 0.99)
14 years	9.6	Ref	20.6	Ref	8.4	Ref		5.0	Ref
15 years	9.5	0.99 (0.88 to 1.09)	10.2	0.96 (0.87 to 1.05)	8.7	1.04 (0.94 to 1.15)		5.2	1.03 (0.92 to 1.14)
16+ years	10.7	1.11 (0.98 to 1.23)	10.5	0.99 (0.88 to 1.10)	9.7	1.16 (1.02 to 1.30)		5.4	1.07 (0.93 to 1.21)
**Times physically attacked in past 12 months**									
Never	8.7	Ref	8.8	Ref	7.2	Ref		4.3	Ref
Once	9.7	1.12 (0.98 to 1.26)	11.5	1.30 (1.15 to 1.45)	8.1	1.12 (1.01 to 1.23)		5.3	1.24 (1.05 to 1.44)
≥ 2 times	11.5	1.33 (1.19 to 1.46)	13.0	1.47 (1.32 to 1.62)	10.2	1.41 (1.29 to 1.54)		6.6	1.54 (1.34 to 1.74)
**Days bullied in past 30 days**									
Never	8.5	Ref	8.9	Ref	6.8	Ref		4.1	Ref
1–2 days	10.5	1.23 (1.11 to 1.36)	11.5	1.30 (1.17 to 1.42)	9.1	1.35 (1.19 to 1.50)		5.8	1.42 (1.23 to 1.61)
≥ 3 days	11.3	1.32 (1.19 to 1.45)	13.0	1.47 (1.31 to 1.62)	11.9	1.76 (1.54 to 1.98)		7.1	1.73 (1.51 to 1.96)
**Went hungry in past 30 days**									
Never	9.5	Ref	10.1	Ref	7.9	Ref		4.7	Ref
Sometimes/rarely	10.0	1.04 (0.93 to 1.16)	10.3	1.01 (0.94 to 1.09)	8.0	1.02 (0.93 to 1.10)		5.0	1.07 (0.97 to 1.17)
Mostly/always	10.1	1.06 (0.92 to 1.20)	12.2	1.21 (1.02 to 1.39)	10.3	1.31 (1.15 to 1.46)		6.6	1.40 (1.14 to 1.65)
**No. of close friends**									
Three or more	9.6	Ref	9.6	Ref	7.5	Ref		4.6	Ref
One or two	9.5	0.99 (0.90 to 1.08)	10.5	1.10 (1.01 to 1.18)	8.3	1.09 (1.00 to 1.19)		5.4	1.16 (1.05 to 1.28)
None	12.0	1.24 (1.08 to 1.40)	14.4	1.50 (1.34 to 1.66)	12.8	1.69 (1.48 to 1.90)		7.2	1.56 (1.30 to 1.82)
**Times felt lonely in past 12 months**									
Never/rarely	8.4	Ref	6.9	Ref	6.2	Ref		3.6	Ref
Sometimes	9.8	1.17 (1.05 to 1.28)	10.3	1.49 (1.35 to 1.63)	8.1	1.30 (1.16 to 1.44)		5.1	1.41 (1.25 to 1.58)
Mostly/always	13.5	1.60 (1.42 to 1.78)	20.8	3.00 (2.70 to 3.31)	14.1	2.27 (2.07 to 2.47)		9.9	2.74 (2.36 to 3.11)
**Parental understanding**									
Mostly/always	8.7	Ref	7.9	Ref	6.6	Ref		3.9	Ref
Sometimes	9.7	1.11 (1.00 to 1.23)	9.1	1.15 (1.03 to 1.27)	8.1	1.20 (1.05 to 1.34)		4.1	1.04 (0.90 to 1.19)
Never/rarely	11.3	1.30 (1.16 to 1.43)	12.8	1.62 (1.49 to 1.75)	14.1	1.45 (1.33 to 1.58)		6.2	1.57 (1.40 to 1.74)
**Smoked cigarettes in past 30 days**									
0 days	9.6	Ref	9.3	Ref	7.5	Ref		4.8	Ref
1–5 days	11.5	1.20 (0.97 to 1.42)	12.8	1.38 (1.22 to 1.54)	11.1	1.48 (1.26 to 1.69)		6.9	1.45 (1.22 to 1.67)
≥ 6 days	11.1	1.15 (0.90 to 1.40)	15.2	1.64 (1.44 to 1.84)	10.3	1.80 (1.50 to 2.11)		6.1	1.29 (1.00 to 1.58)
**Drank alcohol in past 30 days**									
0 days	9.1	Ref	8.2	Ref	7.5	Ref		4.4	Ref
1–2 days	12.0	1.33 (1.15 to 1.50)	12.8	1.57 (1.43 to 1.70)	11.1	1.31 (1.13 to 1.49)		7.3	1.67 (1.40 to 1.93)
≥ 3 days	13.1	1.45 (1.24 to 1.66)	14.8	1.81 (1.61 to 2.01)	13.5	1.50 (1.19 to 1.81)		9.6	2.18 (1.77 to 2.58)
								

CI: confidence interval; Ref: Reference group; RR: Relative risk; WHO: World Health Organization.

^a^ Predicted risks of suicide ideation (with a plan) and RRs were estimated from multivariable logistic regression models that additionally include fixed effects for country and survey year.

Data source: Global School-Based Health Survey.^19^

Regional analyses yielded similar results to country-specific analyses, which identified loneliness, bullying, alcohol use and physical attacks as the most consistent risk factors across countries for both suicidal ideation and ideation with a plan. Of the 32 countries, the RRs were significant for bullying and loneliness in 28 countries, for physical attacks in 26 countries and for lack of parental understanding and alcohol use in 25 countries. Associations between going to bed hungry and smoking and suicide ideation were statistically significant in less than half of the countries. Some determinants showed substantial heterogeneity among countries within the same region in the magnitude of the RRs. For example, there was evidence of substantial heterogeneity within regions for the association between loneliness and suicide ideation (heterogeneity *P*-values < 0.001). By contrast, the association between bullying and suicide ideation was fairly consistent across countries within regions (all heterogeneity *P*-values > 0.05; country-specific results are available from corresponding author).

**Fig. 4:**
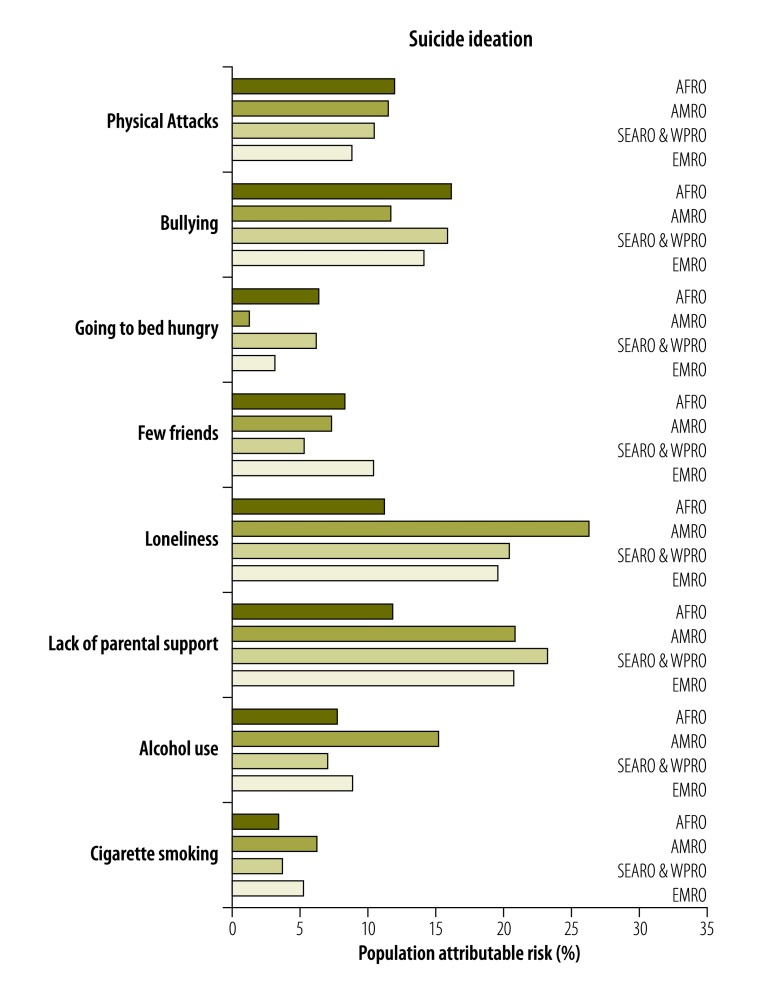
Population attributable risk percentage for determinants of suicide ideation across WHO regions

AFRO: African Region; AMRO: Region of the Americas; EMRO: Eastern Mediterranean Region; SEARO: South-East Asia Region; WPRO: Western Pacific Region.

**Fig. 5:**
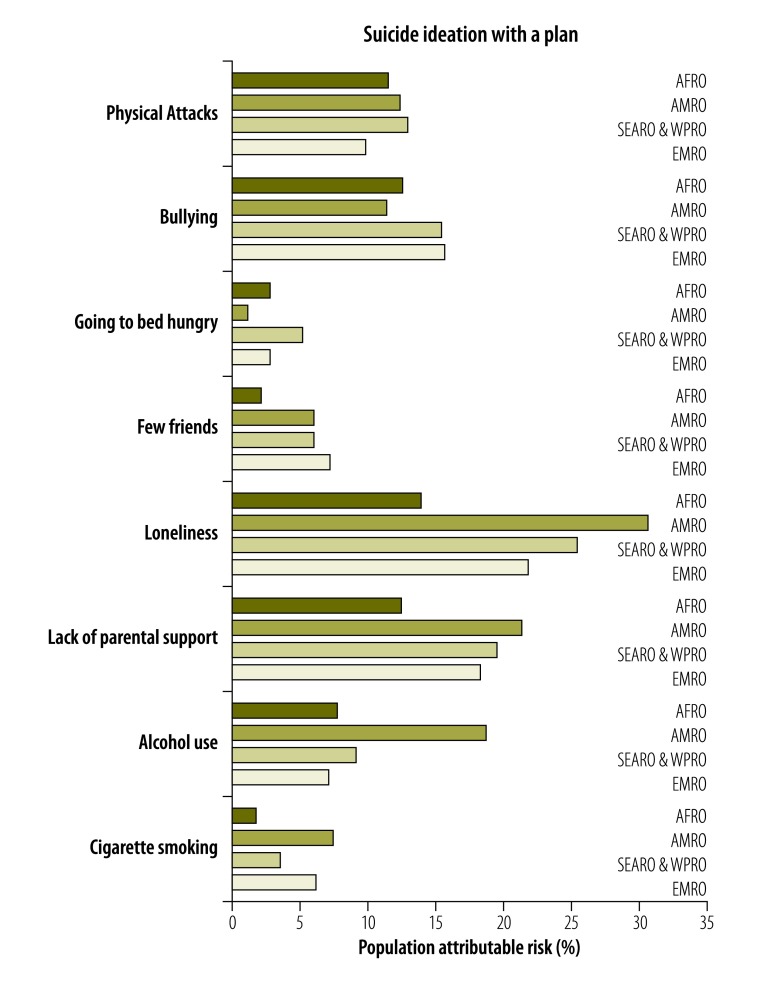
Population attributable risk percentage for determinants of suicide ideation with a plan across WHO regions

AFRO: African Region; AMRO: Region of the Americas; EMRO: Eastern Mediterranean Region; SEARO: South-East Asia Region; WPRO: Western Pacific Region.

PAFs associated with suicide ideation and ideation with a plan across regions are shown in Fig. 4 and Fig. 5. PAF estimates were largest for loneliness and parental support, 26.3% and 20.9%, respectively, in the Region of the Americas. The corresponding values were 20.1% and 22.8% in the South-East Asia and Western Pacific Regions and 18.8% and 19.9% in the Eastern Mediterranean Region. In the African Region, PAF estimates were largest for bullying at 16.2% and for physical attacks at 12.0%. Estimates in the African Region for loneliness and parental support were also considerable at 11.3% and 11.9%, respectively. The PAF estimate for alcohol use in the Americas was 15.3%, approximately twice as high as for the other regions. The PAF for going to bed hungry was 6.4% in the African Region compared to just 1.3% in the Americas. In general, PAF estimates for suicide ideation with a plan found similar results as for suicide ideation.

## Discussion

Our results confirm that adolescent suicidal behaviours are a common problem in low-income and middle-income countries, with prevalence similar to that seen in Europe and North America. However, we found heterogeneity across countries and regions in the prevalence of adolescent suicidal behaviours and in the magnitude of gender differences, which is consistent with previous research.^5^ This variation may in part reflect differences in the meaning of suicidal thoughts and normative attitudes towards suicide across diverse cultural, religious and economic settings.^37^^,^^38^ The higher prevalence of suicidal behaviours among adolescents in African countries may be partly explained by high human immunodeficiency virus (HIV) and acquired immunodeficiency syndrome (AIDS) prevalence, political instability and food insecurity.^27^^,^^39^ The fact that country-specific estimates of adolescent suicide ideation did not correlate strongly with national estimates of suicide deaths is perhaps not surprising given the lack of reliable vital registration data in most countries and the underreporting and misclassification of suicide deaths, particularly those occurring among young people.^2^

The gender differences seen in the Region of the Americas were similar in magnitude to gender differences seen in high-income countries^9^while other regions showed less or no gender disparity. Over the past few decades, Latin America and the Caribbean have made considerable progress reducing gender disparities in school enrolment and labour force participation.^40^ There is evidence that changes in women’s traditional family roles and an increase in the share of women working may initially lead to higher female suicide rates. As gender roles evolve in work and education, adolescent girls might face additional stressors.^41^

Studies have shown a strong association between adverse childhood experiences – such as physical and sexual abuse, parental neglect, bullying – and suicidal behaviours during adolescence and adulthood.^27^^,^^42^ Exposure to these adverse experiences may contribute to suicidal ideation through increasing internalizing behaviours, such as shame, feelings of depression and social isolation, that affect the ability to cope with life stressors.^42^ A longitudinal study from South Africa found a strong and graded association between cumulative exposure to adverse childhood experiences and suicidal behaviour among adolescents aged 10–18 years.^27^ We find consistency across a large and diverse set of countries in the cross-sectional associations between physical violence, bullying victimization and suicide ideation, suggesting that policy efforts aimed at reducing violence and bullying among school-aged youth may help reduce adolescent suicidal behaviours across low- and middle-income countries. However, as in high-income countries, the environmental and social context underlying acts of bullying and physical violence likely differ across cultures, policy regimes and economic zones. For example, physical violence from school staff is an important contributor to the overall burden of youth violence in some countries,^43^ whereas in countries with a high burden of HIV/AIDS, bullying may relate to AIDS-orphanhood and AIDS-related stigma.^44^ Policy approaches will need to consider these local contexts.

Psychological factors such as depression, low self-esteem, hopelessness and weak social relationships are well established correlates of suicidal behaviours among adolescents in high-income countries^9^^,^^45^ and in several low- and middle-income countries.^13^^,^^46^ Although the survey excludes information on common mental disorders, such as depression and generalized anxiety disorder, which are often comorbid with youth suicidal behaviours,^2^^,^^13^ loneliness and social support are important longitudinal predictors of both adolescent depression and suicide ideation.^47^ Across all regions and nearly all countries in our study, psychosocial symptoms, such as loneliness, having few friends and lacking parental support were related to adolescent suicidal behaviours. For countries in the Region of the Americas, Eastern Mediterranean and South-East Asia and Western Pacific Regions, loneliness was the factor most strongly associated with suicide ideation. In the African Region, loneliness was also associated with suicide ideation, but to a lesser extent than in the other regions. Other factors such as bullying, physical attacks and lack of parental control seem to have a stronger influence for suicide ideation in this region.

The strengths of this analysis include the use of standardized measures of suicidal behaviours and risk factors from large number of countries, with most countries having nationally representative samples. In the absence of standardized methods across surveys, cross-national differences in suicidal behaviours and risk factors are more likely to reflect differences in the type of sample, i.e. community-based and school-based, the wording of questions and data collection procedures.^4^ However, there are several limitations that should be kept in mind when interpreting our results. First, given the cross-sectional nature of the data, we were unable to assess temporal relationships between the factors associated with suicide ideation. There is also limited evidence of the validity and reliability of the survey’s measures across culturally diverse settings. Furthermore, in addition to the sensitive nature of questions about suicide, differences in the willingness of students from different cultural backgrounds to report suicidal behaviours and translation of the questionnaires into different languages may also have affected the results.^48^ Finally, restriction of the survey to adolescents currently attending school and present on the day of the survey may have also led to some underreporting of suicidal behaviours.^13^

Here we show similar determinants of adolescent suicidal behaviours across a diverse set of countries, including violence, bullying, lacking friends and alcohol use. Given these risk factors appear quite universal, this information could be used to identify adolescents in school settings who are at increased risk for suicidal thoughts. School-based suicide prevention interventions have been shown to effectively reduce suicide thoughts and attempts among adolescents in rich countries,^49^^,^^50^ however there is a dearth of evidence on effective policies or interventions to reduce youth suicidal behaviours and suicide from low-income and middle-income countries. More research is needed to understand the etiology of suicidal behaviours as well as the types of policies and interventions that can effectively reduce the burden of this critical health challenge.
